# Optimization Of Cancer Treatment Through Overcoming Drug Resistance

**DOI:** 10.31021/jcro.20181107

**Published:** 2018-02-27

**Authors:** Yahya I. Elshimali, Yong Wu, Hussein Khaddour, Yanyuan Wu, Daniela Gradinaru, Hema Sukhija, Seyung S. Chung, Jaydutt V. Vadgama

**Affiliations:** 1 Division of Cancer Research and Training, Department of Internal Medicine, Charles Drew University of Medicine and Science, USA; 2 David Geffen School of Medicine at UCLA, UCLA’s Jonsson Comprehensive Cancer Center, USA; 3 Faculty of Pharmacy, Mazzeh (17th April Street), Damascus University, Damascus, Syria; 4 Carol Davila - University of Medicine and Pharmacy, Faculty of Pharmacy, Department of Biochemistry, Romania

**Keywords:** Drug resistance, Malignancies, Molecular base, Optimal treatment

## Abstract

Cancer Drug resistance is a medical concern that requires extensive research and a thorough understanding in order to overcome. Remarkable achievements related to this field have been accomplished and further work is needed in order to optimize the cure for cancer and serve as the basis for precise medicine with few or no side effects.

## Introduction

Drug Resistance has been reported for most current therapeutic agents against cancer cells, regardless of whether these are chemotherapeutic agents, target therapy or immunotherapy. Heterogeneity of tumor cells is considered an important factor for drug resistance. This heterogeneity is due to renewable small subpopulations of cells called stem-like cancer cells. It is highly possible that different generations of cells exist within one tumor, thus targeting them would result into alternative outcomes as some clones are sensitive and others are resistant to therapeutic agents. Further, tumor cells also have the capability to evolve over time; adding another challenge to treat cancer [1–3].

Initiation of chemo or target therapy for cancer patients depends on the clinical diagnosis, tumor stage, tumor type, molecular characteristics and gene expression.

In early stages, solid tumors (localized) can be cured by surgical excision, but in advanced stages, the chemotherapy, target therapy, immunotherapy, and/or the radiation therapy can be used before or after surgery in order to target the malignant cells that have spread beyond a surgeon’s reach [4]. Cytotoxic drugs are classified into four main categories; DNA Alkylating Agents, Antimetabolites, Intercalating Agents and Mitotic Inhibitors [5]. Chemotherapy drugs are not specific, while target therapies are more specific and have fewer side effects than standard chemotherapy. They were designed to block the receptors that are involved in tumor growth. Targeted therapies include hormone therapies, signal transduction inhibitors, apoptosis inducers, gene expression modulators, angiogenesis inhibitors, and toxin delivery molecules [6], whereas immunotherapies insvolve three general categories: checkpoint inhibitors, cytokines, Interferon and cancer vaccines [7]. The optimal application for cancer drugs can be determined by the genetic signature of every individual tumor case, thus identifying the best appropriate drug combination to be used and to avoid unnecessary toxicity. Although these treatments brought great success by prolonging the survival period, understanding the resistance to these agents will definitely optimize the cure for cancer and serve as the basis for precise medicine with less cytotoxicity [3].

## Mechanisms of Resistance to Cancer Drugs

There are two types of drug resistance, one occurs prior to drug treatment known as primary or innate resistance, and the other develops over time post exposure to a given therapeutic agent known as acquired resistance; both being associated with genetic and epigenetic changes that occur within a cancer cell [8].

On the other hand, mechanisms of resistance to cancer drugs are divided into two broad categories: cellular mechanisms and non-cellular mechanisms. Cellular mechanisms address tumor cell-autonomous signaling pathways, insensitivity to natural growth arrest signals, abolishment of cell contact inhibition, the ability to evade apoptosis, and the role of the tumor microenvironment, whereas non-cellular mechanisms are related to pharmacological response [1]. Factors that are associated with tumor cell-intrinsic modifications include genetic alterations, chromatin modifications, enrichment of cancer stem/initiating cells, loss of cell polarity, alterations in cell-cell and cell-matrix adhesion, and deregulated receptor kinase signaling, which all together support detachment, migration and invasion of tumor cells which negatively influence the response to chemotherapy.

The interactions between tumor cells and non-transformed cells, such as stromal, endothelial, and immune response cells are other factors that manipulate tumorigenic events and responses to chemo, target, or immuno drugs [9–12]

### Cellular mechanisms

#### Genetic mutations that promote cancer drug resistance

Mutations that result in over-expression of growth factors or down-regulation of tumor suppressor genes will enhance cell proliferation and cancer drug resistance. Hence, pathways affected by these mutations include:

#### Activation of RAS/RAF/MEK/ERK signaling pathway

The RAS/RAF/MEK/ERK pathway is implicated in growth-factor mediated cell proliferation, differentiation, and cell death. Activation of this pathway will result in resistance to chemo drugs and target therapy.

Inducting signals of this pathway could be due to overexpression of EGFR or mutations of tyrosine kinases receptors (RTK) that turn them into an active state leading to the activation of downstream signaling cascade and promoting cancer cellular survival, proliferation, and up regulation of fatty acid synthase [13–16].

Mutation of KRAS may lead to constitutive activation of the signal transduction pathway, which is associated with development of resistance to anti-EGFR monoclonal antibody with agents like centuximab or panitumumab. B-Raf mutations have been frequently detected in melanoma and thyroid cancers, and resistance to chemotherapy was observed in ectopic activation of Raf doxorubicin treated tumors or paclitaxel treated breast cancer cells. Interestingly, inhibition of ERK pathway increases tumor cell resistance to ADM (Adriamycin) and GEM Gemcitabine [17–20]. More than 80% of patients with non-small-cell lung cancers (NSCLC) are over expressing EGFR1 in their tumor tissues indicating poor prognosis and resistance to chemotherapy, and only 10% of these patients respond to EGFR1-TKI (tyrosine kinase inhibitor) therapy. In order to overcome such resistance, a combination of target therapy against both EGFR and MEK is indicated and similar combination against EGFR and c-Met was also addressed in patients with lung fibrosis and who are expressing abundantly type I collagen in the tumor mass [21–24].

Other drugs that act on this pathway are Raf kinase inhibitors (sorafenib, encorafenib) and MEK inhibitors (cobimetinib, selumetinib and trametinib) [24].

#### Mutations observed in PTEN/PI3K/AKT/MTOR pathway

The PI3K/Akt/mTOR pathway is a central regulator in cancer cell proliferation, tumorigenesis, and metastasis. This pathway is comprised of three main driving molecules: PI3K (phosphoinositide 3-kinase), AKT, and MTOR (mammalian target of rapamycin) [25–28].

PTEN (phosphatase and tensin homologue deleted from chromosome 10) is a dual protein and lipid phosphatase that is commonly mutated in many human malignancies. PETN was originally identified as a negative regulator of the PI3K leading to inactivation of AKT and MTOR signaling. Loss of PTEN results in reduced dephosphorylation of PIP_3_ (phosphoinositide 3,4,5-trisphosphate), which allows PI3K to phosphorylate PIP_2_ (phosphatidylinositol 4,5-bisphosphate) and enhances levels of PIP_3_. PIP_3_ induction activates AKt and causes increased cell size, cell proliferation, cell migration, cell survival, and resistance to chemotherapy. Loss of function of PTEN or constitutive activation in the gene encoding the catalytic subunit of PI3K (PIK3CA) results in resistance to Trastuzumab [27–31]. PTEN loss/mutation often co-exists with mutations in BRAF and KRAS, and preclinical studies suggested PTEN deficiency is associated with resistance to the EGFR inhibitors (cetuximab, gefitinib and erlotinib) for treatment of colorectal and lung cancer [28–32]. PTEN function and expression are modulated by germline and somatic PTEN mutations, genomic deletion, epigenetic silencing, post-transcriptional regulation, post-translational regulation, and protein-protein interactions [27–33]. On the other hand, the alterations on both pathways RAS/RAF/MEK/ERK and PTEN/PI3K/AKT/MTOR seem to have significant influence in melanoma progression, survival, and chemo-resistance [30–33].

AKT has been reported as a mediator in immune escape by activating the expression of immune checkpoint receptor programmed death-ligand 1 (PD-L1) in cancer cells avoiding their elimination by T lymphocytes [33].

#### Activation of NF-kB and STATs

Transcriptional factors of the NF-κB family and STAT3 are commonly regulating the expression of various genes associated with cell-cycle progression, angiogenesis, immune responses, apoptosis, metabolism, and cancer [34].

STAT3 can bind to and dissociate NF-kB:IkB complex, prolonging NF-kB retention in the nucleus, and promoting target gene activation [34–35]. The microenvironment of malignant cells, including immune cells and stromal cells, play an important role in the interaction between STAT3 and NF-κB through different kinds of cytokines. Thus, IKKβ inhibitors may slow down tumor growth and augment susceptibility to other therapeutic agents through preventing the expression of key tumor promoting cytokines. In addition, activated NF-κB has been identified as a key mechanism for the resistance to cisplatin. On the other hand, treatment of cells with AKT-NF-κB inhibitor abrogated the increased NF-κB activity in murine models results in sensitizing them to cisplatin induced apoptosis. However, disruption of either NF-κB or STAT3 signaling does not lead to cell death, therefore their inhibitors should be combined with cancer-specific cytotoxic drugs. In order to avoid the associated toxicity with theses inhibitors, it is desirable to consider more specific strategies that target NF-κB- and STAT3-dependent tumor promoting cytokines, such as TNF-α, IL-6 and IL-23. Also, a natural agent such as resveratrol can inhibit constitutive activation of both NF-kappa B and STAT3 resulting in down-regulation of cell proliferation [34–41].

#### Inactivation of Tumor Suppressor Genes (TSGs)

Tumor suppressor genes maintain genomic stability, integrity of cell cycle, and prevent tumor development. Mutations of TSGs have been implicated in primary cancer development and in therapeutic resistance.

#### BRCA1 (Breast-Cancer Susceptibility Gene 1)

BRCA1 functions as a tumor suppressor gene by regulating transcription, cell cycle checkpoint, and DNA repair. BRCA1 is frequently mutated in inherited breast and ovarian cancers though somatic mutations rarely reported in sporadic cases. Nevertheless, down-regulation of BRCA1 occurs more often with epigenetic modifications [42].

BRCA1 expression predicts the outcome of breast, lung, and ovarian cancer treatment with DNA-damage-based therapy and depending on the type of chemotherapeutics, BRCA1 can have either a positive or negative role in mediating chemo-sensitivity. When BRCA1 is inactivated by mutations, deletions, or down-regulation, cancer cells will no longer be able to repair the drug-induced DNA damage and will therefore trigger apoptosis, which explains why loss of BRCA1 sensitizes cancer cells to DNA damaging agents such as cisplatin and PARP [poly (ADP-ribose) polymerase] inhibitors. Similar findings have been applied to basal-like breast cancer which is characterized by being triple negative and aggressive [42–44]. Defects in DNA repair is a promising therapeutic target as BRCA alterations are found in 11 to 42% of these tumors, with a frequency varying according to family history and ethnicity. The oral PARP inhibitors exploit this deficiency through a synthetic lethality and are considered as promising anticancer therapies, especially in patients harboring BRCA1 or BRCA 2 mutations [44].

On the other hand, in response to microtubule damage induced by anti-microtubule agents such as paclitaxel or docetaxel, BRCA1 is activated to induce mitotic arrest and apoptosis by transcriptionally activating the spindle assembly checkpoint protein MAD2 (myoadenylate deaminase 2), which results in the activation of the JNK pathway via direct interaction with the JNK–MEKK3/ERK complex. Therefore, when BRCA1 is lost, the cancer cells will no longer activate the mitotic spindle checkpoint protein MAD2, and subsequent activation of the pro-apoptotic JNK pathway will cause resistance to the anti-microtubule agent [44–45].

In epithelial ovarian cancer, low let-7e (21-nucleotide regulatory microRNA) [46] leads to activation of BRCA1 and Rad 51 (339-amino acid protein that plays a major role in homologous recombination of DNA during double strand break repair) [47]. With the subsequent enhancement of double strand break repair causing cisplatin-resistance, re-expression of let-7e might be an effective strategy for overcoming chemo-resistance [45].

#### (Retinoblastoma gene)

Over-expression of Rb gene is considered an important marker for predicting chemotherapeutic response, Yet, several studies revealed conflicting evidence regarding the loss of Rb gene or not yielding chemo resistance [43].

#### TP53 Gene

TP53 is one of the most well-known tumor suppressor genes. Mutant p53 not only functions as a tumor suppressor, but can also exert tumor-promoting effects. Hence, the majority of human cancers show the inactivation of the p53 pathway.

Loss of p53 causes drug resistance due to down-regulation of pro-apoptotic genes such as PUMA (p53 up-regulated modulator of apoptosis), Bax, Bid, and Noxa.

On the other hand, PTEN can have tumor promoting effects on cells expressing mutant p53; therefore p53 status should be determined when PTEN is involved in a pathway of therapeutic interest [43–49].

#### The Hippo Tumor Suppressor Pathway

The Hippo pathway is an onco-suppressor signaling cascade that plays a major role in the control of cell growth, tissue homoeostasis, organ size, stem cell renewal, and the response of cancer cells to chemotherapy [50].

It is composed of three components: cell surface upstream regulators including cell adhesion molecules and cell polarity complexes; a kinase cascade comprising two serine-threonine kinases with regulators and adaptors; and a downstream target, a transcription coactivator [49,50]. In this pathway, the Mst1/2 (serine/threonine kinases/Hippo in Drosophila) and LATS1/2 (large tumor suppressor) together with the adaptor protein SAV1(Salvador homologue 1) and hMOB1 are the core players that transmit signals from upstream tumor suppressors molecules (Fat4, RASSF1A, Kibra, Merlin, hEx, and hWW45) to the downstream targets and tightly restrict the activities of homologous oncoproteins YAP (Yes kinase-associated protein) and (TAZ) (transcriptional co-activator with PDZ-binding motif) [50,51].

Hippo pathway negatively regulates the co-activator to restrict cell proliferation and to promote cell death. Hence, dysregulation of this pathway leads to aberrant activation of the transcription co-activator YAP and TAZ that contributes to tumorigenesis in several tissues. The localization of the MST1/2 and LATS1/2, whether it is cytoplasmic or nuclear, may impact the efficacy of the neoadjuvant therapy in breast cancer; being protective when expressed in the cytoplasm of tumor cells and in tumor-infiltrating lymphocytes. The dysfunction of this pathway is frequently detected in human cancers and correlates with a poor prognosis [52,53].

#### Molecules that affect cell cycle

Cell cycle progression is regulated by various complicated pathways that control every stage of the cell cycle (G0/G1, S and G2/M phases). Dysfunction or mutation of any regulator in each pathway may cause abnormal cell proliferation and may result in malignancy [54]. Invasion occurs primarily in a G_1_/G_0_ cell cycle-arrested state, and expression of pro invasive genes driving epithelial to mesenchymal transition and F-actin cytoskeletal reorganization are associated with this cell cycle state [55–57]. Changes in the activity of cyclin-dependent kinase inhibitors CDK (cyclin-dependent kinase) (P21 and p27, INK4 family, P16, P21) and their target not only mediate the decision to enter or exit the cell cycle, but also may be critical to acquiring an invasive phenotype [58,59].

Targeting some molecules through these pathways could synergize or increase the effect of chemo drugs, but drug resistance can still occur as an evolving event through the course of treatment.

Of these molecules, these are important for cell cycle involved in chemo resistance CASC1 (CAncer Susceptibility Candidate 1), TRIM69 (Tripartite motif containing 69), FOXO1 (Forkhead box protein O1), Kin17 (DNA/RNA-binding protein KIN17), and (Short transient receptor potential channel 5) TrpC5 CASC1 is essential for microtubule polymerization of spindle assembly checkpoint and is frequently co-amplified with KRAS in lung tumors while TRIM 69 is important for formation of a bipolar spindle. Study shows that RNAi-mediated attenuation of CASC1 or TRIM69 inhibits tumor growth *in vivo* and thus it can be a target to overcome paclitaxel induced resistance.

FOXM1 facilitates DNA repair through regulating direct transcriptional target EXO1 (exonuclease 1) to prevent cancer cells from cisplatin-mediated apoptosis as observed in ovarian cancers [56–60].

Kin17 is a conserved nuclear protein that participates in DNA damage repair, DNA replication, and cell proliferation. Kin17 protein expression was up-regulated in patients exhibiting chemo resistance in oral squamous cell carcinoma.

Adriamycin-resistant human breast cancer cells possessed numerous TrpC5 containing extracellular vehicles (EVs) on the cell surface. Up-regulated TrpC5 accumulated in EVs is responsible for EV formation and EV trapping of chemotherapeutic drugs. Thus, using TrpC5-containing EVs could be used as a diagnostic biomarker for chemo resistant breast cancer [61].

### Oxidative stress and tumor hypoxia

#### Oxidative stress

The interaction between tumor cells and incubated microenvironment, i.e. stromal cells, surrounded stroma, neo vasculatures, oxygen, and nutrient supplements are essential factors for tumor growth, invasion, and metastases.

Tumor cells gain fundamental adaptation to a new environment by utilizing energy and using glucose through aerobic and anaerobic pathways, revealing more capability to adapt to the new stressful or normal conditions than normal counterpart cells and eventually adding further means for drug resistance [62,63].

“Sorcin” is an important protein that is up-regulated under the stressful conditions of the endoplasmic reticulum (ER). It is a 22-kDa calcium-binding protein that regulates epithelial-mesenchymal transition and cancer stem cells (CSCs), partly through E-cadherin and vascular endothelial growth factor expression. Scorin enhances the accumulation of Ca (2+) in the endoplasmic reticulum (ER) in order to prevent ER stress and render cancer cells resistant to chemotherapeutic agents [64].

In contrast, RNAi-mediated silencing of sorcin activated caspase-3, caspase-12, and GRP78/BiP, triggers apoptosis through the mitochondrial pathway. Experiments on human colorectal cancer cells (CRC) showed over-expression of sorcin as an adaptive mechanism to prevent ER stress and escape apoptosis prompted by chemotherapeutic agents. Nevertheless, Scorin was found to induce low levels of paclitaxel resistance in human ovarian and breast cancer cells and is associated with multidrug resistance in leukemia cells as well [65].

On the other hand, over-expression of CARMA3 {CARD (Caspase recruitment domain) recruited membrane associated protein 3} in human epithelial ovarian cancer is also associated with cisplatin resistance [54].

The Nrf2 {NF-E2-related factor 2) antioxidant response pathway is the primary cellular defense against the cytotoxic effects of oxidative stress. Nrf2 increases the expression of several antioxidant enzymes and its overexpression in cancer is associated with negative roles against chemo agents such as cisplatin. Several drugs that stimulate the Nrf2 pathway are being studied for treatment of diseases that are caused by oxidative stress as well [66,67].

Kawata et. al. [68] analyzed the immunohistochemical results of anti-oxidant response genes on prostate cancer cells {NRF2 (nuclear factor erythroid 2–related factor 2) and NQO1(NAD(P)H Quinone Dehydrogenase 1)} and found that it was more up-regulated after hormone ablation in prostate carcinoma samples after ADT (androgen deprivation therapy) than in untreated specimens or in murine prostate glands after castration, suggesting that ADT induces cellular senescence processes accompanied by secretory phenotypes and anti-oxidant responses in prostate cancer cells. These cellular changes may be attractive targets for preventing endocrine resistance in prostate cancer as observed in treatment with the anti-oxidant agent NAC (N-acetyl-cysteine), which significantly suppressed SA-β-Gal (senescence-associated-β-galactosidase) activity in androgen-sensitive human prostate cancer cells (LNCaP) [67,69].

#### Tumor hypoxia

The center of a solid tumor is highly hypoxic due to poor blood circulation and this hypoxia is considered to be a major contributor to drug resistance. Under severe hypoxia, gene expression of ubiquitously expressed key enzymes and transporters of folate metabolism, as well as nucleoside homeostasis are down regulated.

The activated hypoxia induced factors (HIFs) may induce the expression of numerous gene products that can be targets for therapy and ultimately decrease the drug resistance [69,70]. Examples of these targets are Pluripotency-associated transcription factors (Oct-3/4, Nanog and Sox-2), CXCR4 (C-X-C chemokine receptor type 4), Snail, Twist, VEGF, and Micro RNAs [71].

On the other hand, Antifolates have a crucial role in the treatment of various cancers by inhibiting key enzymes in purine and thymidylate biosynthesis. Resistance to antifolates, which is associated with suppression of folate metabolism, may result from the failure of antifolates to induce DNA damage under hypoxia that is attributed to hypoxia-induced cell cycle arrest rather than a general anti-apoptotic mechanism [72,73].

#### The Role of (ABC) Transporter Proteins in Drug Resistance

A Family of energy-dependent multi-drug transporters known as ATP-binding cassette transporter proteins (ABC) plays remarkable roles in chemo drug resentence [74–76]. The role of transport proteins is decisive to maintain cellular homeostasis. They have normal chemo protective functions in cells throughout the body against metabolites, small molecules, drugs, and endogenous toxins.

In cancers, the cells utilize certain transporter proteins to efflux chemotherapeutic agents out of the cells in order to support their survival. However, several studies have suggested that these transporters may block the entry of chemotherapeutic agents as well. Nevertheless, chemotherapeutic drugs need to enter and remain inside the cells in order to be effective [77]. The ABC proteins transport various molecules across the cellular membranes, and of these proteins are P-glycoprotein 1 (permeability glycoprotein, Pgp) known as MDR1 (multidrug resistance protein 1) and MRP2 (multidrug resistance-associated protein 2). Hence, over-expression of P-glycoprotein (P-gp) encoded by MDR1 gene is one of the major causes of drug resistance [78,79].

Vascular cell adhesion molecule-1 or CD106 (VCAM-1) is a transmembrane glycoprotein similar to ABC transporter protein that activates TGFβ1 or IL-6 mediating cell migration and facilitates leukocyte adhesion, leukocyte trans-endothelial migration, and cell activation by binding to integrin VLA-1 (α4β1) on T lymphocytes, VCAM-1 helps in recruitment of inflammatory cells toward tumor microenvironment and its expression is rapidly induced by proinflammatory cytokines such as TNF-α, IL-6 and TGF-β1, Over-expression of VCAM-1 along with CD44 and ABCG2 are associated with resistance to doxorubicin and cisplatin in breast cancer subjects [80–82].

MRP1, unlike MDR1, transports negatively charged natural-product drugs that have been modified by glutathione conjugation, glucosylation, sulfation, and glucuronylation. However, in some cases, cotransport of glutathione with positively charged drugs such as vinblastine may occur and the alteration of the cellular membrane transporters causes increased energy-dependent efflux of hydrophobic cytotoxic drugs keeping intracellular concentrations below the cell-killing threshold [74,78,82].

Variety of cancer cells showed resistant to colchicine, vinblastine, doxorubicin, vinca alkaloids, etoposide, paclitaxel, and other small molecules that are considered to be substrates for this transport system [78,82,83].

On the other hand, acquisition of drug resistance after chemotherapy is associated with increased P-gp levels that occur via specific molecular mechanisms, such as gene rearrangement, Likewise, expression of P-gp in some tumors predicts poor response to chemotherapy with drugs that are transported by P-gp [82–84].

Studies in multiple myeloma patients revealed an association between cyclin D1 gene amplification and disease severity, poor prognosis, and increased expression of MDR [85–87].

In osteosarcoma patients, the expression of the transcription factor Trps1 (trichorhinophalangeal syndrome I) is directly correlated with expression MDR1/P-gp (93). ABCB6-mediated MDR (ATP-binding cassette sub-family B member 6) is clinically relevant in some malignancies and highly expressed in breast cancer patients with detectable minimal residual disease and in patients with hepatocellular carcinoma, as opposed to a healthy liver. Also, ABCB6 is up-regulated in cell lines treated with arsenite, camptothecin, or cisplatin and cell lines of primary and secondary melanoma [88,89].

The correlation of point mutations in class III β-tubulin (TUBB3) and the prominent overexpression of ATP-binding cassette P-glycoprotein (ABCB1) have been protruding mechanisms of resistance to microtubule disruptors such as paclitaxel (PTX) for many cancers, These findings highlight the control of the TUBB3 response to ABCB1 genetic suppressors as a mechanism to reverse the profuse development of multidrug resistance in cancer.

Furthermore, the interaction between MRP1, FOXO3a, TUBB3, and PI3K/Akt signaling has been linked with doxorubicin resistance [90].

Finally, resistance to methotrexate (which is toxic folate analogs), or other nucleoside analogs commonly occurs by mutation of one or both of the folate transporters or specific mutation to the nucleoside transporters [91].

#### Multidrug Efflux Pumps/Resistance and Cancer Stem Like Cells (CSC)

Cancer cellular heterogeneity represents a variety of cell types as well as epigenetic differences amongst the cancer cells themselves. Cancer stem like cells (CSC) is one component of this heterogeneity, which was defined by the American Association for Cancer Research (AACR) meeting in 2006 as “Self-renewable cells with pluripotency capacity to generate heterogeneous lineages of cancer cells that comprise the tumor.” However, the four major characteristics of CSCs are: self-renewing capacity; differentiation capacity; tumor-initiating capacity; and metastatic potential.

Paradoxically, the ABC efflux pumps afford protection to cancer stem cells (CSCs), shielding them from the adverse effects of chemotherapeutic insult. Hence, CSCs retains the essential property of self-protection through the activity of multiple drug resistance (MDR) transporters [92].

Current chemo-therapies of malignant tumors may eliminate the total, or near the total of the mass of malignant cells, but apparently it fails to eradicate or target the CSCs which are believed to be the cause for relapse or metastasis.

The CSC resistance may result from gene mutation, which could bepresent prior to chemotherapy, or as a result of environmental differences.

Therefore, understanding the mechanisms that regulate some traits of CSCs may help design efficient strategies to overcome chemo-resistance [93].

#### Altered target enzyme (e.g. mutated topoisomerase II)

Topoisomerase II (topo II) is a ubiquitous essential nuclear enzyme that is essential for cell survival. Topo II regulates DNA topology, DNA replication, relegates DNA fragments, modifying the linking number of a DNA loop and promotes chromosome disentanglement [94,95].

Topoisomerase II is a target of alkaloid and anthracycline agents and varieties of mutations in this gene have been associated with the development of drug resistance [96].

Topo II can be targeted by small molecules that are divided into two classes: inhibitors and poisons. The inhibitors of topoisomerase II include HU-331, ICRF-187, ICRF-193, and mitindomide. These molecules are noncompetitive inhibitors that reduce ATPase activity. Poisons of type II topoisomerases (have been extensively used as both anticancer and antibacterial therapies) include etoposide, novobiocin, quinolones (including ciprofloxacin), and teniposide. These small molecules target the DNA-protein complex leading to increased cleavage and inhibiting DNA relegation. Alteration in the enzyme function, structure, or production due to Top II mutation will impede the interaction with inhibitors and result in resistance to chemotherapy [97–99].

In triple negative breast cancer (TNBC), targeted therapies are not effective, and chemo agents currently are the main modality available for systemic therapy. Thus, the anthracyclines may be effective in treatment of TNBC as far as the topo II is not mutated [100].

#### Expression of T-box transcription factor T (also known as brachyury)

Brachyury may attenuate cell cycle progression, enabling tumor cells to become less susceptible to radiation and chemotherapy in human carcinomas. Brachyury is a molecule frequently detected in human cancers but seldom found in normal adult tissue and has recently been characterized as a driver of the epithelial-to-mesenchymal switch of human carcinomas [100,101]. Chromatin immunoprecipitation and luciferase reporter assays revealed that Brachyury binds to a half T-box consensus site located within the promoter region of the p21 gene, indicating a potential mechanism for the observed therapeutic resistance associated with Brachyury expression [101].

Studies showed the attempts to reduce or knockdown Brachyury will reduce invasiveness, chemoresistance and radioresistance of CSCs *in vivo.* Therefore, Brachyury knockdown may be a useful therapeutic tool for sensitizing CSCs to conventional chemo-radiotherapy.

Also, *in vitro* and *in vivo* human lung carcinoma cells with higher levels of Brachyury divide at slower rates than those with lower levels of Brachyury, a phenomenon associated with marked down-regulation of cyclin D1, phosphorylated Rb, and CDKN1A [101,102].

### Non-Cellular Mechanisms

Non-cellular drug resistance is linked to the extracellular influences and is associated with unique characteristic of the tumor environment. Tumor regions that are deficient in nutrients and oxygen may reduce drug access, drug accumulation, and prevent tumor cells from cytotoxicity.

Myofibroblasts and extracellular matrix (ECM) proteins contribute to the anti-apoptotic protection of tumor cells. So, cellular adhesion molecules (e.g. L1CAM or CD44), chemokines (e.g. CXCL12), integrins, and other ECM receptors that are involved in direct and indirect interactions between tumor cells and their microenvironment have been identified as proper molecular targets to overcome chemoresistance [103].

#### Micro RNA and Resistant To Chemotherapy

Each micro RNA is complementary or partially complementary to one or more of mRNA molecules and its main function is to regulate the gene expression. MicroRNAs (miRNAs) are a novel class of endogenous short, non-coding RNA molecules of which the mature form is about 22 nucleotides in length. MiRNAs are counted as master regulators of gene expression by either cleaving or binding directly to its 3′-UTR region [104,105].

Several studies revealed the role of MiRNAs in chemo resistance of various malignancies and modulate multiple signaling pathways adding another mechanism of multi-drug resistance so that even subtle changes in miRNAs expression can cause significant changes in disease progression and in cancer outcomes. Depending on the cellular function of miRNAs targets, these molecules could be either an oncogene or a tumor suppressor gene [105].

Moreover, miRNAs can have opposite effects towards the same anticancer agent in different tumor types. However, the association between the effects of miRNAs and the methylation on gene expression during the progression of chemoresistance can alter the potency of various anticancer agents inversely in the same cancer cell, suggesting that the relationship between the function of miRNAs and drug resistance is highly complex [106].

#### Epigenetic modifications

Epigenetic modification plays a critical role during the development of acquired chemoresistance and is important in evaluating the potential application for biomarkers in cancer diagnosis as well. Epigenetics refers to the functional changes of the genome caused by methylation and/or histone post-translational modification that alters gene expression without altering the underlying DNA sequence [106,107].

DNA methylation frequently occurs in repeated sequences through the covalent addition of a methyl (CH_3_) group at the 5-carbon of the cytosine ring resulting in 5-methylcytosine [106–108].

UHRF1 (Ubiquitin-like containing PHD and RING finger domains 1) is one master regulator gene/protein in epigenetics which coordinates DNA methylation and histone modifications, which also mediates repair of damaged DNA that makes cancer cells resistant toward cytocidal drugs [109–110].

UHRF1 protein binds to specific DNA sequences and recruits DNMT1 (DNA methyltransferase 1) to regulate chromatin structure and gene expression. Its expression peaks at the late G1 phase and continues during G2 and M phases of the cell cycle. It plays a major role in the G1/S transition by regulating topoisomerase II alpha and retinoblastoma gene expressions and functions in the p53-dependent DNA damage checkpoint [111].

Therefore, the functional domains of UHRF1 utilize epigenetic inhibitory effects on TSGs including p16 with subsequent inhibition of the apoptotic pathways. It is also notable that UHRF1 regulates other TSG as observed with CDX2, CDKN2A, RUNX3, FOXO4, PPARG, BRCA1 and PLM in gastric cancer, SOCS3 and 3OST2 in endometrial carcinoma, RB1 in Jurkat and osteosarcoma cells, as well as BRCA1 in cancer breast cell lines. UHRF1 is overexpressed in colorectal cancer (CRC), non-small cell lung cancer (NSCLC) and gastric cancer. Furthermore, its high expression level was associated with an increase in the expression of DNMT1, DNMT3A, and DNMT3B, and correlated with tumor progression and drug resistance. Hence, any agent that decreases or suppresses the expression of UHRF1/DNMT1, as observed with natural anti-cancer drug, epigallocatechin-3-gallate (EGCG), will result in cell cycle G1/S arrest and apoptosis.

In certain human cancer cell lines, the expression at the mRNA level, protein kinase activity and tumor cell anti-apoptotic activity and resistance were related to the methylation status of the caspase-8 gene promoter. Combination therapy coupled with demethylation reagents may overcome therapeutic resistance in certain malignancies [110–112].

#### Attempts to Overcome Resistance

In order to achieve a significant therapeutic outcome in cancers, the malignant cells need to lose their chemo protective features mediated by MRP or MDR-1, as well as enhancing the apoptotic rate of these cells.

Numerous factors could influence the ability of chemo drugs to kill cancer cells. This includes, but is not limited to, drug pharmacokinetics and metabolism; changes in microenvironment; genetic and epigenetic modifications; DNA repair genes; tumor suppressor genes; multidrug-resistance genes; apoptotic related genes; and abundant growth factors. Therefore, understanding the underlying cause of resistance is the initial step in the journey of overcoming this challenge in cancer treatment [113].

1. As mentioned, MDR is operated by extrusion pumps, a group of ATP-binding cassette (ABC) drug transporters which include P-glycoprotein (P-gp). The P-gp overexpression in cancer cells has become a therapeutic target for bypassing MDR. One approach that has already been applied in the clinical setting has shown potentially in overcoming MDR by encapsulation of the P-gp substrate drugs in liposomes or nanoparticles [114–116].
2. Developing anticancer drugs that are not substrates for P-gp are not susceptible to extrusion from P-gp overexpressing tumor cells. Data regarding taxanes, tesetaxel (DJ-927), and milataxel (MAC-321) shows that these drugs are poor substrates for P-gp and have demonstrated superior antitumor activity compared to docetaxel in vitro and in vivo [117–118].
3. Overcoming resistance may be accomplished by molecules that inhibit the activity of P-gp and the efflux transporter such as telatinib or silibinin. These are natural compounds isolated from milk thistle seed extracts that inhibit ABCG2 efflux transporter activity and increase the effectiveness of drugs inside tumor cells [118,119].
4. Inducing cell apoptosis by chemotherapy is one approach used to kill cancer cells, however, this process is often inhibited in tumor cells due to overexpression of the anti-apoptotic protein Bcl-2 or the decreased expression of pro-apoptotic proteins, such as Fas, Bax, or cysteine proteases (caspase proteins) [120–123].
5. Overexpression of protein tyrosine kinases (PTKs), such as EGFR, HER2, and IGFR activate different cell signaling pathways that include PI3/AKT, NIFκB, STAT3, and ERK1/2, which also lead to aberrant expression apoptosis related proteins in cancer cells that are the major causes of cancer cells resistance to chemotherapies. Therefore, target therapy against specific tyrosine kinases will overcome such resistance [124]. Over years, target therapies have been developed and shown to be promising in the clinical world. Trastuzumab, an antibody that targets and binds with high affinity to the cell surface bounds HER2 receptors and prevents receptor activation [125]. Interestingly, in some conditions, resistance may evolve during time against target therapy and could be due to different mutations at a target location, multiple break points of translocations, or dysfunction in phosphorylation of proteins substrates. Hence, finding new small molecules, specific inhibitors, or combinations between target therapy and chemotherapy may prolong the progression time of breast cancer, and significantly improve survival, as opposed to chemotherapy alone [126].
  On the other hand, blocking the activation of EGFR, an antibody that selectively binds EGFR-by Cetuximab, has shown to improve the response rate of 5-Fu in patients with metastatic colorectal cancer who initially failed 5-FU-based therapy [127,128].
6. DNA methylation is an important mechanism that may lead to aberrant expression of apoptosis related genes. Then, combination of chemotherapies with agents that can reverse the methylation status is promising to overcome drug resistance [129,130].
7. Combined chemotherapy and immunotherapy: It is promising that immunotherapy may reform the treatment of cancer by inducing, augmenting or suppressing immune responses against cancer cells, which also include monoclonal antibodies, cancer vaccines, and inhibitors of immune checkpoints such as anti PD-1/PDL-1 [131,132].
8. Gene knockout using antisense molecules or CRISPR/Cas9 gene editing has shown to be an effective method for blocking drug resistance genes [133].
9. Combination of autophagy inhibitors such as Chloroquine and its derivative with cytotoxic drugs is attracting more attention in cancer therapy. However, further work is needed to understand fully the functional relevance of autophagy within the tumor microenvironment and the interaction with other signaling pathways related to cancer drug resistance [134].
10. Advancing technology for using small interfering RNA (siRNA) may create a novel treatment modality in gene-specific silencing that significantly suppresses gene expression at the messenger RNA (mRNA) and prohibit protein production [135–138]. Nevertheless, many miRs functioning as TSGs have also been shown to regulate drug sensitivity. For example, down-regulation of Let-7, miR-34, or miR-181 increases chemosensitivity, whereas down-regulation of miR-127 causes chemoresistance [139]. Overexpression of miR-125a-5p increases sensitivity to drugs, whereas overexpression of miR-15-5p is associated with drug resistance.
  The growing list of these miRNAs and their roles for mediating a chemotherapeutic response suggests their importance in regulating tumorigenesis and preventing cancer drug resistance [140,141]. Therefore, chemical modifications of these molecules and the use of viral vectors or nanoparticles in treatment of cancer patients may overcome this challenge.
11. Development of small molecules target the histone modifiers such as KDM4B, may provide an opportunity to enhance the efficacy of standard chemotherapies or overcome drug resistances [142–148].

## Conclusion

The advancement in molecular biology and in bioinformatics allows us to establish “molecular signatures” for cancer patients and identify individuals who will benefit from particular therapies.

Application of new laboratory testing such as liquid biopsy by measuring cell free RNA or cell free DNA, as well as performing sequencing of cancer genomes from FFPE tissue or from plasma, will enhance our capability to select the optimal drugs and to avoid ineffective treatment in order to accomplish the best clinical outcome.
